# On interpretation and task selection: the sub-component hypothesis of cognitive noise effects

**DOI:** 10.3389/fpsyg.2014.01598

**Published:** 2015-01-15

**Authors:** Patrik Sörqvist

**Affiliations:** Department of Building, Energy, and Environmental Engineering, University of GävleGävle, Sweden

**Keywords:** noise, cognition, task selection, interpretation, attentional capture, process impurity

## Abstract

It is often argued that the effects of noise on a “complex ability” (e.g., reading, writing, calculation) can be explained by the impairment noise causes to some ability (e.g., working memory) upon which the complex ability depends. Because of this, tasks that measure “sub-component abilities” (i.e., those abilities upon which complex abilities depend) are often deemed sufficient in cognitive noise studies, even when the primary interest is to understand the effects of noise as they arise in applied settings (e.g., offices and schools). This approach can be called the “sub-component hypothesis of cognitive noise effects.” The present paper discusses two things that are troublesome for this approach: difficulties with interpretation and generalizability. A complete understanding of the effects of noise on complex abilities requires studying the complex ability itself. Cognitive noise researches must, therefore, employ tasks that mimic the tasks that are actually carried out in the applied setting to which the results are intended to be generalized. Tasks that measure “sub-component abilities” may be complementary, but should not be given priority in applied cognitive research.

## INTRODUCTION

It is often argued that complex behavior such as reading and writing depends on working memory—the ability to hold and manipulate information in mind—or executive functions such as inhibition or updating (Baddeley, 2007; Carretti et al., 2009). For example, when reading, people encode orthographic information, transform it into a phonological code and maintain, and manipulate that information in working memory to integrate it with long-term memory representations and produce comprehension. Working memory can, therefore, be viewed as an ability that supports the complex behavior (reading) although it is not the only ability involved in the complex behavior. The term “sub-component ability” will be used to refer to abilities such as working memory and executive functions upon which “complex abilities” depend.

Noise impairs performance on tasks that are designed to measure sub-component abilities, such as short-term/working memory (Haapakangas et al., 2011; Schlittmeier et al., 2011; Tremblay et al., 2012; Hughes, 2014), executive functions (Sörqvist et al., 2010; Jahncke et al., 2011), and retrieval from semantic memory (Jahncke, 2012; Jones et al., 2012). Noise also impairs performance on tasks that are designed to measure complex abilities, such as reading (Cauchard et al., 2012), writing (Ransdell and Gilroy, 2001; Keus van de Poll et al., 2014), proofreading (Venetjoki et al., 2006), and memory of written discourse (Bell et al., 2008). There has been a fundamental error in my way of thinking about the effects of noise on complex abilities, based in part on poor interpretation of findings such as the reliable relationship between individual differences in working memory capacity and reading comprehension (Just and Carpenter, 1992; McVay and Kane, 2012). The error can be called the “sub-component hypothesis of cognitive noise effects”: the idea that the effects of noise on complex abilities can be studied, quantified and understood by solely investigating the effects of noise on sub-component abilities. The purpose here is to discuss where I went wrong and to help others in the same situation.

## THE SUB-COMPONENT HYPOTHESIS OF COGNITIVE NOISE EFFECTS

It is often argued that the effects of noise on complex cognitive abilities can be explained as a result of an impairment of some more basic, supporting cognitive ability. For example, the effects of background speech on executive functions or working memory are sometimes referred to as an explanation of the effects of background speech on reading comprehension (Sörqvist et al., 2010). Naturally, this is just an example that is relatively easy to understand, which is why it will be returned to throughout this paper. The same discussion applies to any other complex cognitive ability (e.g., the ability to calculate, write, and speak) that depends upon sub-component abilities (e.g., inhibition, working memory, shifting, updating, etc.).

The “sub-component hypothesis of cognitive noise effects” is sometimes used as a reason to employ tasks that are designed to measure sub-component abilities, even when the research question is about the effects of noise on performance as they arise in applied settings (e.g., the office environment). For example, studying the effects of noise on short-term memory of semantic information is sometimes believed to reveal how noise impairs performance in the office environment (Jahncke et al., 2013). A task of particular interest is the classic visual-verbal serial recall task. In this task, participants study sequences of visually presented items (e.g., “l b m t q d p”) and are asked to recall the sequence, in the given order, immediately after presentation. Serial recall is very sensitive to noise effects (Ellermeier and Zimmer, 1997), which is why the task is highly suitable for detailed analyses of the mechanisms underpinning auditory distraction (cf. Hughes, 2014). However, the task is also often employed in cognitive noise studies that aim to understand the effects of noise as they arise in applied settings such as office environments (Perham et al., 2009; Schlittmeier and Hellbrück, 2009; Haapakangas et al., 2011) and traffic control rooms (Tremblay et al., 2012). So even if the type of task that is carried out in the “real-world” environment is much more complex (e.g., word processed writing, tracking airplanes on a visual display), it is deemed sufficient to measure sub-component abilities to understand and quantify the effects of noise in applied settings. This approach is encumbered with various conceptual difficulties that emerge largely from two general problems of interpretation: the “process impurity” problem and the propensity of sound to capture attention (Sörqvist, 2014).

### THE PROCESS IMPURITY PROBLEM

A cognitive task measures many different things, not necessarily only what it is designed to measure. Consider, for example, the classic visual-verbal serial recall task described above. This task requires many cognitive operations such as maintenance of items in short-term memory, rehearsal, and updating between trials (old sequences must be forgotten/suppressed so as to not interfere, proactively, with new sequences). Hence, the task is not “process pure.” All cognitive tasks are, to some extent, “process impure” (Surprenant and Neath, 2009) and what they measure depends largely on the cognitive operations and processes the participants choose to carry out while completing the task. What the participants do—cognitively—when they undertake the task is more important for interpretation than what the task was intentionally designed to measure. For example, the same “short-term memory” task—in terms of materials and procedure—can render susceptible to distraction by noise under some strategy instructions but not under other strategy instructions (Perham et al., 2007).

The “process impurity” problem has various consequences for interpretation of noise effects (Sörqvist, 2014), including the “sub-component hypothesis of cognitive noise effects.” Consider again the effects of background speech on reading comprehension (Martin et al., 1988). A task that is designed to measure reading comprehension is not process pure. It measures, for instance, the ability to maintain information in mind over the short term, the ability to retrieve appropriate as opposed to inappropriate information from long-term memory to interpret the text, the ability to inhibit or suppress inappropriate text interpretations, and so on. Part of this overlaps, arguably, with the cognitive processes involved in tasks that are designed to measure working memory, such as the classic visual-verbal serial recall task. For example, both tasks require maintenance of information in mind over the short term and suppression of outdated information. Conversely, part of the processes does not overlap. Whilst reading comprehension requires integration of new information with information presented far back, to understand how the discourse unfolds, the ability to integrate information is not tapped by the classic serial recall task (e.g., Perfetti and Goldman, 1976).

The problem that arises with the “sub-component hypothesis of cognitive noise effects” is that the effects of noise on tasks designed to measure sub-component abilities may be functionally different from the effects of noise on complex abilities. That is, the cognitive process that is impaired by noise, and hence the reason why task performance is reduced, may be categorically different in the context of the task that measures the sub-component ability, on the one hand, and in the context of the task that measures the complex ability, on the other. The most crucial point to be made here is that experiments, only involving tasks designed to measure sub-component abilities, because this approach is deemed sufficient to understand the effects of noise as they arise in intellectual work environments, run a substantial risk of being misleading. The effects of noise on tasks that are designed to measure sub-component abilities cannot, straightforwardly, be generalized to “real-world” environments, because the effects of noise on complex abilities—the ones that are carried out in intellectual work environments—could be very different both in function and in magnitude. Part of the problem could, potentially, be attenuated by careful task requirement manipulations, in order to identify the exact mechanism behind the impairment (Sörqvist, 2014), but the attention capture problem makes interpretation and generalizability even more problematic.

### THE ATTENTION CAPTURE PROBLEM

One way by which noise can impair cognitive performance is by capturing attention. For example, if participants do the classic visual-verbal serial recall task against a background of spoken sentences, performance drops drastically when the participants’ own name is embedded within the sentences, compared to a control name (Röer et al., 2013). The reason for this is probably that the detection of one’s own name calls for attention, causing a reallocation of the locus-of-attention away from the to-be-recalled items. Attentional capture produces disruption to the cognitive task by interrupting the cognitive activity, not by corrupting the cognitive processes or cognitive structures (Hughes, 2014). This is a fundamentally important point that has to be appreciated when the effects of noise on cognitive performance are interpreted (Sörqvist, 2014). Otherwise, one may confuse the cognitive ability/structure with the operationalization of that cognitive ability/structure and interpret effects of noise on task performance (e.g., memory of written prose) as reflecting a corrupted cognitive ability/structure (e.g., episodic memory) rather than as an interruption to ongoing cognitive processes.

A number of factors modulate the propensity of sound to capture attention. One of those is task difficulty. Sound loses its ability to capture attention when the task is difficult (Hughes et al., 2013; Halin et al., 2014a,b). The reasons for this appear to be that the locus-of-attention becomes more steadfast (Hughes et al., 2013) and that the (neural) processing of the sound is more constrained (Sörqvist et al., 2012a,b) when the task is difficult. The fact that the propensity of sound to capture attention depends on various factors, including task difficulty, has consequences for the “sub-component hypothesis of cognitive noise effects.” Assume that attentional capture is responsible for the effects of noise on a reading comprehension task as well as for the effects of noise on a working memory task. It would be wrong to interpret this as suggesting that the effect of noise on reading comprehension is explained by a disrupted working memory. The accurate interpretation is that cognitive processes are interrupted in both cases. Moreover, effect sizes can hardly be generalized. As different tasks vary in difficulty, and difficulty modulates the magnitude of noise effects, it is very likely that the effects of noise on tasks designed to measure complex abilities (e.g., proofreading, writing, and reading) and effects of noise on tasks designed to measure sub-component abilities (e.g., serial recall, executive function tasks) are different in magnitude.

### EMPIRICAL EVIDENCE AGAINST THE “SUB-COMPONENT HYPOTHESIS OF COGNITIVE NOISE EFFECTS”

In an experiment from a few years back, we asked participants to undertake the *number updating task* and a reading comprehension task, both in silence and against a background of speech (Sörqvist et al., 2010). The updating task is designed to measure the executive function called “updating” (i.e., the ability to exchange information in working memory, by encoding new information and suppressing no-longer wanted information) although it certainly measures many other things as well (e.g., rehearsal). As several studies have found positive correlations between performance on updating tasks and on reading comprehension tasks (Carretti et al., 2009) we had the idea that the effects of speech on reading comprehension could be explained by the impairment caused by noise to updating processes. This was tested with a mediation analysis whereby the difference scores for the two tasks, respectively, were obtained by calculating the difference between the silent condition and the background speech condition, and then testing the correlation between the difference scores. This mediation analysis did not support the sub-component hypothesis. The negative conclusion may be premature (Sætrevik and Sörqvist, 2014), but our study (Sörqvist et al., 2010) did not provide empirical evidence for the “sub-component hypothesis of cognitive noise effects.” And even if it had, interpreting the mediation analysis as if the effect of noise on reading comprehension is the result of impaired updating processes would be highly problematic for the reasons described above (process impurity and attentional capture).

## CONCLUSION

It is impossible to fully understand—let alone to quantify—the effects of noise on complex abilities based on the effects of noise on tasks that are designed to measure sub-component abilities. It is, hence, necessary to study the effects of noise on complex abilities rather than stopping with sub-component processes. Cognitive noise researchers should consider the problems (i.e., the process impurity problem and the consequences of attention capture) associated with the sub-component hypothesis when selecting tasks for their investigations and interpreting their findings, especially those who attempt to understand how and why noise effects arise in applied settings such as schools, offices, and other environments for intellectual work. In particular, generalizations to applied situations from effects of noise on tasks that measure “sub-component abilities” should be made with caution.

## Conflict of Interest Statement

The author declares that the research was conducted in the absence of any commercial or financial relationships that could be construed as a potential conflict of interest.
